# Can left ventricular endocardial surface roughness be measured by fractal dimension on fast gradient echo sequences?

**DOI:** 10.1186/1532-429X-18-S1-P41

**Published:** 2016-01-27

**Authors:** Filip Zemrak, Gabriella Captur, Patricia Feuchter, Charlotte Manisty, Mark Westwood, Saidi A Mohiddin, James C Moon, Steffen E Petersen

**Affiliations:** 1 Centre of Advanced Cardiovascular Imaging, Queen Mary University of London, London, UK; 2 Cardiovascular Imaging, Barts Heart Centre, London, UK

## Background

Left ventricular (LV) fractal dimension (FD) on cardiac magnetic resonance (CMR) balanced steady-state free precession (SSFP) cines has already proven to be useful in making a diagnosis of non-compaction cardiomyopathy. Normal FD values for SSFP cines have been established (Captur 2015).

Fast gradient echo (fGRE) sequences have different contrast between blood pool and myocardium, which affects measurement of FD. However, if FD could be measured on fGRE it would allow for retrospective analysis of scans acquired before implementation of SSFP or in patients with devices that underwent fGRE.

The aim of this study was to establish a correlation between FD measured on SSFP and fGRE sequences in the same subjects.

## Methods

Twelve participants of Barts Cardiovascular Registry who underwent cardiac magnetic resonance (CMR) imaging with 1.5T scanners for various clinical reasons had cine short axis stack (SAX) acquired with both SSFP and fGRE sequences.

We measured FD in 57 corresponding (positioned in the same LV location) SAX cine slices on SSFP and fGRE using dedicated MATLAB script and an Osirix "LVFractalAnalysisFilter" plugin written in Objective-C with equivalent architecture. The same region-based level-set segmentation and fractal procedure was implemented. Briefly: the scale parameter specifying the size of the neighbourhood (sigma) was set to 2%; size of the initial contour was set to 6 × 9 pixels; and maximum initial grid-size for fractal analysis was 45% of the original size of the region of interest estimated through the application of a scaled bounding box.

We used Pearson's correlation coefficients and two-way random single measures of consistency intraclass correlation coefficients (ICC) to compare FD from SSFP and fGRE, first for values derived in MATLAB and then in Osirix.

## Results

FD on fGRE was around 6.5% larger than on SSFP in MATLAB and 4.3% in Osirix (p < 0.001 and p < 0.05). There was a good agreement between the FD derived from SSFP and fGRE sequence in both MATLAB and Osirix tools (ICC = 0.65 and ICC = 0.72, respectively).

Detailed results are presented in the table and the figure.

## Conclusions

This proof-of-principle analysis shows a significant correlation between FD on SSFP and fGRE sequences. FD measured on fGRE may have utility as a research tool and further ground-truth validation of the method is encouraged.

**Figure 1:**
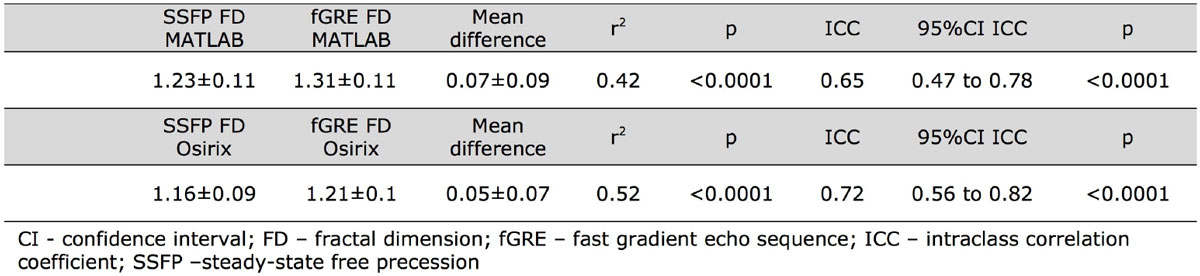
**Comparison of left ventricular fractal dimension from fGRE and SSFP sequences measured in MATLAB and Osirix tools - Pearson's correlation and intraclass correlation coefficients ICC**.

**Figure 2:**
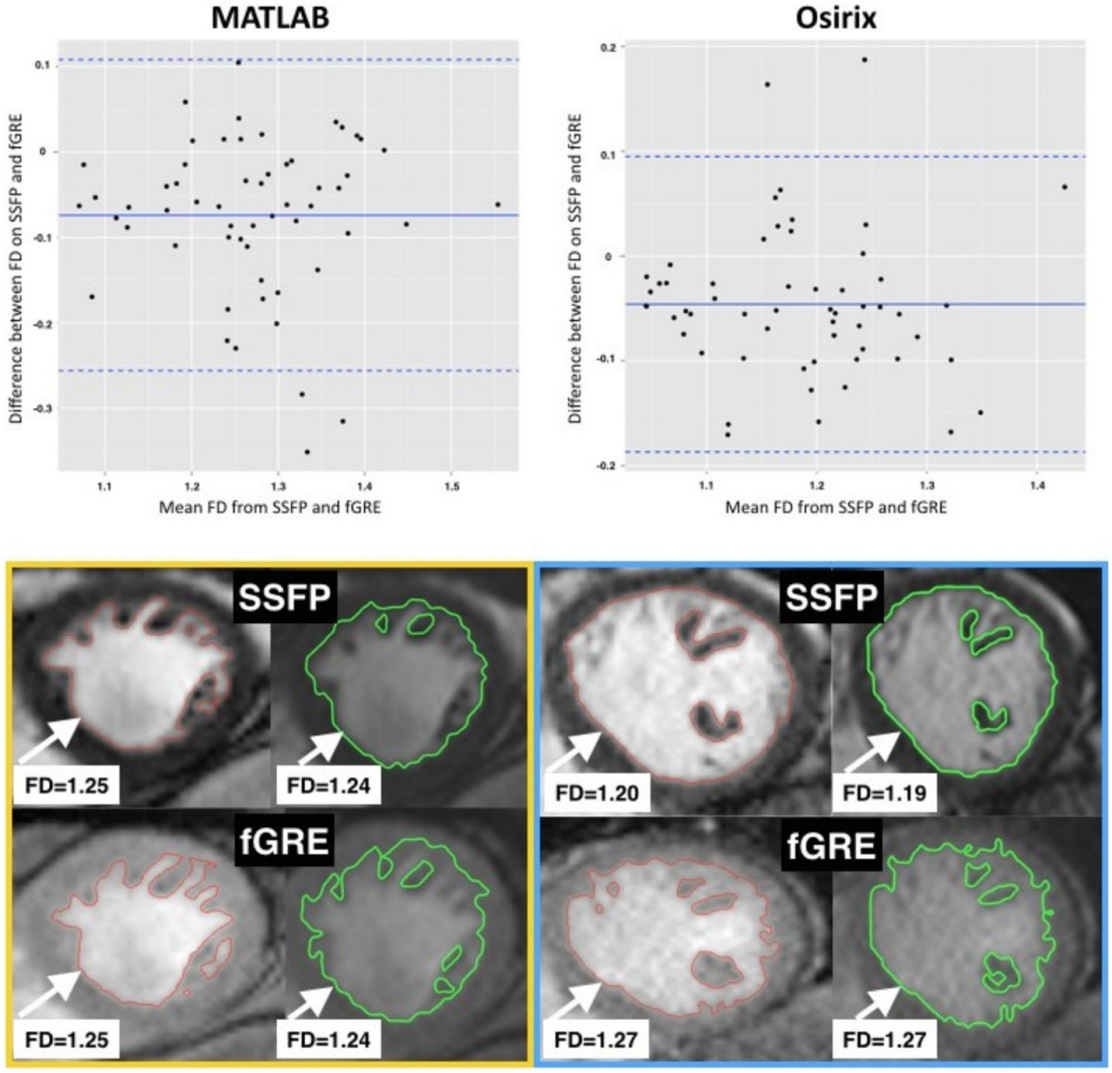
**Bland-Altman plots showing differences between FD from SSFP and fGRE sequences in the corresponding SAX slices measured in MATLAB and Osirix**. Yellow box: an example of FD analysis of the same SAX slice in a subject with greatest agreement between SSFP (upper panels) and fGRE (bottom panels) in both MATLAB (red line) and Osirix (green line). Blue box: an example of the largest difference in FD between SSFP and fGRE on the same SAX slice.

